# Embodied language and early motor restriction: evidence from children with obstetric brachial plexus palsy and arthrogryposis

**DOI:** 10.3389/fpsyg.2026.1832623

**Published:** 2026-05-19

**Authors:** Dzerassa Kadieva, Evgeny Blagoveshchensky, Olga Agranovich, Andriy Myachykov, Anna Shestakova

**Affiliations:** 1 Centre for Cognition and Decision Making, Institute for Cognitive Neuroscience, HSE University, Moscow, Russia; 2 Laboratory of Behavioural Neurodynamics, St. Petersburg State University, St. Petersburg, Russia; 3 The Turner Scientific Research Institute for Children’s Orthopaedics, St. Petersburg, Russia; 4 Cognitive Health and Intelligence Centre, Institute for Cognitive Neuroscience, HSE University, Moscow, Russia; 5 Centre for Cognitive and Brain Sciences, University of Macau, Taipa, Macau SAR, China

**Keywords:** action semantics, arthrogryposis multiplex congenita, cognitive skills, embodied cognition, motor disorder, obstetric brachial plexus palsy

## Abstract

Embodied and embedded cognition (EEC) theory proposes that language and cognitive development emerge from bodily interactions with the environment, yet empirical tests of this claim in clinical developmental populations remain rare. This mini-review synthesizes behavioral, electrophysiological, and structural neuroimaging evidence from children with serious early motor disorders—obstetric brachial plexus palsy and arthrogryposis multiplex congenita—which restrict upper limb movement from birth or before, providing a unique opportunity to test EEC predictions in a motor-restricted population. The results reveal a gradient of cognitive and linguistic alterations: from domain-specific deficits in action-verb semantics and verbal fluency, to broader impairments in memory, categorical reasoning, and naturalistic neural processing. Based on these multimodal findings, we propose that early sensorimotor restriction does not only affect motor systems but may shape the neurodevelopmental trajectory of language and other distributed cognitive architectures through mechanisms of embodied grounding.

## Introduction

Over the past three decades, embodied cognition has become an influential theoretical framework in cognitive science, motivating research across cognitive psychology, pedagogy, linguistics, and developmental science. Embodied cognition as a concept encompasses numerous and, in some cases, contradictory ideas, however, they all share a common ground of dynamic cognition that is not and cannot be governed by the brain alone. Currently, embodied cognition is often coupled with the notion of embeddedness. The embodied embedded cognition (EEC) model argues that behavior and cognition emerge from bodily interactions with the environment within the internal and external constraints that those interactions imply (Barsalou, 2008). It is through the body that we get to know what is possible, and our thinking follows. The environment scaffolds mental processes, while the brain plays a coordinating role in these interactions, as opposed to dictating them from above (Thelen and Smith, 1994). This results in an interconnected and rather nonlinear system of body, brain, and environment.

**Figure 1:**
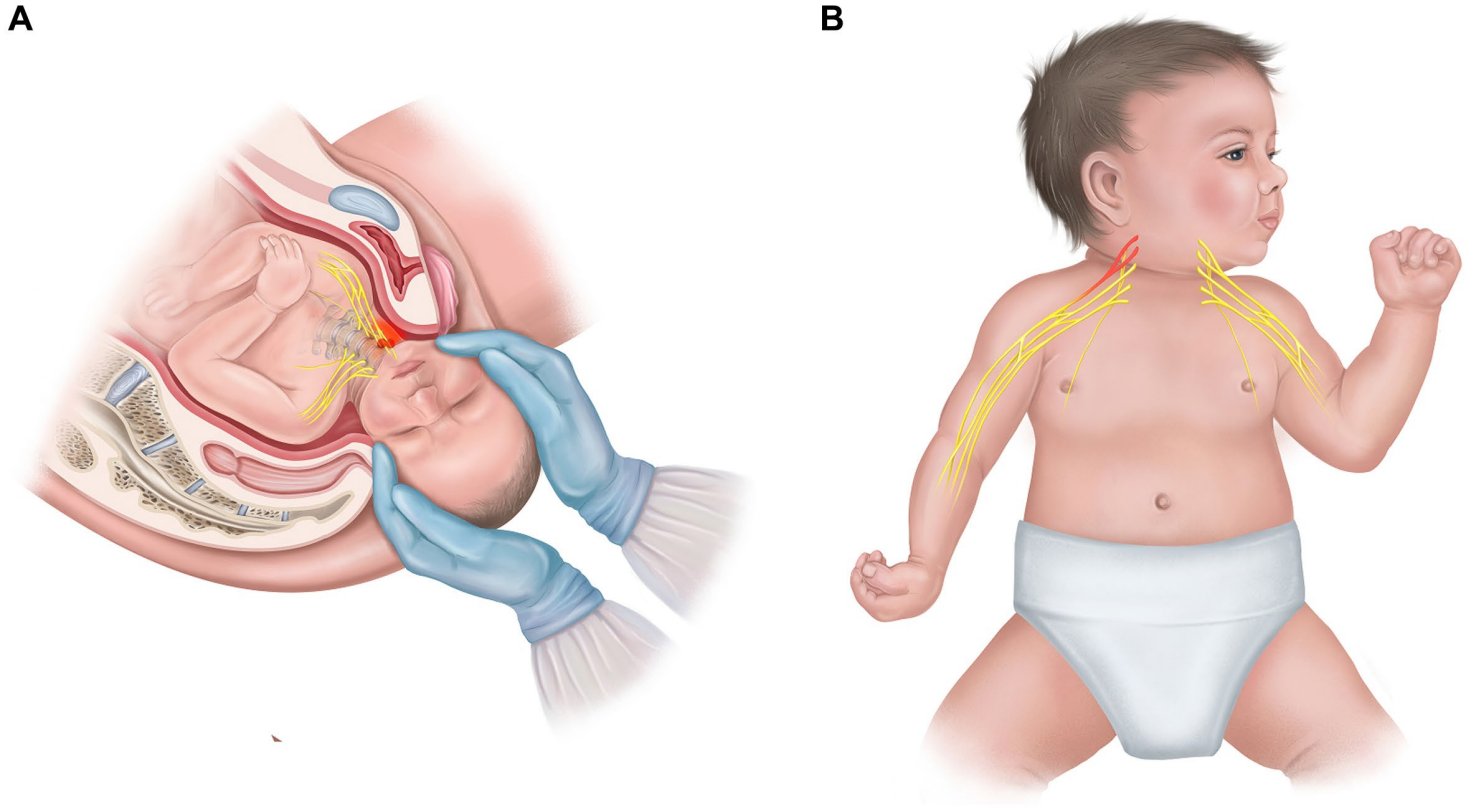
**(A)** Mechanism of obstetric brachial plexus palsy (OBPP): traumatic stretching of the brachial plexus nerve roots during delivery, with the site of injury highlighted in red. **(B)** Upper-type OBPP. Affected nerve roots (C5-C6) result in unilateral paralysis of shoulder abduction and elbow flexion, while the contralateral arm remains intact.

Despite substantial philosophical discourse and numerous laboratory demonstrations, empirical validation of EEC in developmental populations remains surprisingly rare. To test the limits and predictions of EEC theory, it might be useful to examine cases where the “body” variable is systematically altered prenatally or perinatally. This allows us to observe how altered sensorimotor experience shapes the developing cognitive architecture. Over the past half-decade, our research team has investigated a clinical group consisting of children with obstetric brachial plexus palsy (OBPP) and arthrogryposis multiplex congenita (AMC). These conditions cause motor restrictions due to peripheral neuromuscular injury (OBPP) or congenital limitation (AMC). OBPP results from brachial plexus trauma during delivery, causing unilateral upper limb paralysis (Socolovsky et al., 2016; Figure 1A). The extent of impairment depends on which nerve roots are affected, with upper trunk injuries (C5-C6) being most common and producing deficits in shoulder abduction and elbow flexion (Figure 1B). AMC is a congenital condition characterized by reduced or absent fetal movement in utero (fetal akinesia) and affected motor neurons, usually producing bilateral limb contractures and muscle weakness (Kalampokas et al., 2012). Our studies focused on cases involving proximal arm muscles with functional impairment of the upper limb requiring similar treatment approaches. However, even within this selection, the conditions differ in their underlying mechanisms. OBPP involves peripheral nerve injury often accompanied by sensory disruption (Martínez-Carlón-Reina et al., 2024), while AMC is primarily musculoskeletal, with intact sensory pathways in most cases (Hall, 2014). OBPP is unilateral by definition, whereas AMC may be bilateral. AMC represents prenatal developmental deprivation, while OBPP represents post-traumatic loss of function imposed on a brain that developed a typical prenatal sensorimotor baseline.

Despite the acknowledged clinical differences, young patients with both OBPP and AMC represent a unique methodological opportunity for testing EEC predictions. Both conditions in our studies shared critical features: (1) restriction of upper limb movement present at birth, though in AMC this restriction originates prenatally through fetal akinesia; (2) anatomical specificity to upper limbs; (3) static, nonprogressive clinical course. The anatomical specificity allows for precise, falsifiable predictions about which cognitive domains are likely to be affected and which should remain relatively preserved. Therefore, we suggest that the cognitive deficits observed in children with OBPP and AMC might be explained by the altered ways in which these children engage with their early physical and social surroundings. In this review, we synthesize behavioral, electrophysiological, and structural neuroimaging evidence to examine whether early peripheral motor restriction is associated with altered cognitive development, with a focus on action semantics, memory, executive functions, and naturalistic information processing. We discuss both EEC-based interpretations and alternative explanatory frameworks.

## From motor restriction to action semantics

A central prediction of EEC is that the conceptual understanding of actions is not represented as abstract, amodal symbols but is grounded in the same sensorimotor circuits used to perceive and perform those actions (Gallese and Sinigaglia, 2011). If action concepts are indeed based on sensorimotor experience, then motor impairment should produce fairly specific deficits in action-semantic processing. Children with OBPP and AMC provide an extreme test of this prediction. We assessed action semantics through language tasks and automatic brain responses to action words, probing both explicit retrieval and implicit perceptual representations of motor-related concepts.

Our behavioral evidence showed that motor restriction can disrupt the production and perception of action-related speech. Children with OBPP and AMC, with intact vocal tracts, demonstrated impaired verbal fluency, producing significantly fewer words compared with their age-matched controls (Koriakina et al., 2025). These deficits were most pronounced with action and occupation words, further followed by weaker results in action naming tasks (naming actions presented in pictures). Additionally, patients showed significantly lower results in semantic association tests, specifically in action-object coupling (e.g., switching the lights and a light bulb). General vocabulary and grammatical processing did not differ significantly, demonstrating preserved language competence in non-motor domains. If motor restriction simply caused general developmental delay, we would expect uniform impairments across all language tasks. Instead, the selective concentration of deficits in the motor-semantic domain strongly supports the hypothesis that the inability to physically perform movements with the affected limb may impede the formation of robust semantic representations for those specific concepts.

The deficits further extend to semantic associations between objects (e.g., a lemon squeezer and a lemon), suggesting that impairment is not limited to retrieving isolated action words (Koriakina et al., 2025). This finding aligns with affordance theory (Gibson, 1979), which proposes that objects are represented in terms of the actions they afford. Without the ability to physically interact with objects using the hands, the affordance-based organization of object concepts may develop poorly. While these behavioral deficits demonstrate motor-semantic coupling, they could also reflect disruptions at multiple processing levels, from early semantic access to late response selection. Thus, we examined automatic, pre-attentive brain responses to action words (Pulvermüller, 2005; Hauk et al., 2004) to determine whether motor restriction alters the representational structure of action concepts themselves. Using mismatch negativity (MMN) paradigms (Näätänen et al., 2007), we measured EEG responses to action words in both patients and age-matched controls (Bredikhin et al., 2023). The MMN is an event-related potential component that occurs automatically when a deviant stimulus violates an established regularity in a sequence of standard stimuli, typically manifesting as increased negativity between 150 and 250 milliseconds post-stimulus, capturing local prediction error signals or mismatches when category-specific regularities are violated (Näätänen et al., 2007; Dehaene et al., 2015).

Children with OBPP and AMC showed a delayed and reduced MMN response to hand-related action verbs and normal response to leg-related action verbs and pseudowords (Bredikhin et al., 2023). This pattern leads to several important conclusions. Our MMN findings rule out a general deficit in action-word processing, since leg-related action words showed normal responses. Basic auditory discrimination, phonological processing, or attention appear not to be affected either, since phonologically similar pseudowords also showed normal responses. The selective impairment for hand-action words might imply that motor limitation can alter the underlying structure of hand action concepts themselves, consistent with the idea that sensorimotor experience helps build semantics.

Pulvermüller and his colleagues have provided extensive evidence that action-word meanings are grounded in distributed neuronal circuits linking language areas with somatotopically organized motor and premotor cortex (Pulvermüller, 2001, 2005, 2018; Hauk et al., 2004; Shtyrov et al., 2004, 2014), with further studies demonstrating that such category-specific semantic organization emerges naturally from correlated sensorimotor experience during word learning (Garagnani and Pulvermüller, 2016; Tomasello et al., 2017). In children with upper limb motor restrictions, the motor component of this correlated activity is absent or severely reduced during the critical period of word learning. Our MMN results also argue against simple accounts based purely on undifferentiated statistical learning, since it fails to explain why automatic pre-attentive perceptual processing is altered only for hand-related action words. As a limitation to the MMN study it should be stated that we did not conduct a direct comparison between OBPP and AMC subgroups.

## Cognitive effects beyond action semantics

Language deficits extended beyond action semantics. Children with OBPP and AMC showed reduced verbal fluency for animals, impaired object naming across multiple categories, and difficulties with object-object semantic associations (Koriakina et al., 2025). Here, three non-mutually-exclusive explanations deserve consideration.

The first, grounded in the representational EEC approach, proposes that semantic categories in typically developing children are organized around multimodal clusters including perceptual properties, functional relationships, and action-based information (Unger et al., 2016). Consider the category of animals: while animals certainly possess distinctive visual features, they are also encoded through characteristic movement patterns (walk, run, fly, or swim) and through typical interaction affordances (pet, feed, or play) (Booth and Waxman, 2002). Similarly, object concepts are organized around the actions they afford: a hammer is represented not only through its visual appearance but also through grasping and striking actions (Gibson, 1979; Borghi, 2004). When motor experience is restricted from birth, this action-based organization may be weakened, compromising semantic network formation even for concepts with no obvious motor content.

The second, based on the works by Iverson and colleagues on interactional EEC, proposes that children typically manipulate objects while caregivers provide labels and action descriptions, building multimodal associations among motor experience, perceptual features, and words (Iverson, 2010, 2021; West and Iverson, 2017). Motor restrictions may disrupt these triadic interactions that normally ground semantic knowledge. Through thousands of such episodes, the child builds integrated semantic representations that bind together visual appearance, tactile properties, motor actions, object functions, and linguistic labels. Those episodes determine which objects become salient, generate rich haptic and proprioceptive information, and trigger the caregiver’s linguistic input in a synchronized manner. Children with OBPP and especially AMC have fewer opportunities for independent object manipulation, thereby reducing these learning interactions from the very beginning. As a result, their semantic networks may develop with poorer and weaker representational connections, explaining why children with motor restrictions show not only specific action-semantic deficits but also broader impairments in semantic fluency, category fluency, and associative processing across multiple domains.

The third explanation invokes the “embedded” aspect of developmental vulnerability arising from negative psychosocial factors. Children with physical disabilities frequently face extended periods of hospitalization, reduced peer interaction, parental overprotection, and social exclusion (Blagovechtchenski et al., 2023). These factors carry documented independent effects on cognitive and language development. Pediatric hospitalization is associated with delays in cognitive outcomes (Fardell et al., 2023), while reduced social participation limits children’s access to linguistically diverse contexts that support vocabulary development broadly across semantic domains, not exclusively motor-semantic ones (Hoff, 2006; Mashburn et al., 2009).

It should be notes that no significant differences in verbal fluency or semantic association performance were found between children diagnosed with AMC and those with OBPP (Koriakina et al., 2025). However, children with bilateral limb impairment performed significantly worse on the action-object semantic association task than those with unilateral impairment (Koriakina et al., 2025), providing preliminary support for the laterality effects.

## Broader cognitive and neural consequences

The impact of early motor restriction extends beyond semantic processing, affecting core executive and memory functions that are not obviously related to motor abilities. Both auditory working memory (digit span) and visual working memory (picture recall) were significantly impaired in children with OBPP and AMC as compared with healthy controls (Blagovechtchenski et al., 2023; Koriakina et al., 2021). Notably, we found no difference between AMC and OBPP groups, suggesting both conditions produce enough motor restriction to affect memory. However, we cannot yet say whether this reflects a threshold or a continuous relationship. Such memory impairments do not seem like adaptations or compensations. They are more plausibly interpreted as part of a broader developmental reorganization. Through structural MRI, children with OBPP showed reduced gray matter volume in bilateral anterior hippocampus and left cerebellar Crus I as compared with healthy controls (Kadieva et al., 2024). Given the hippocampus’s role in multimodal memory formation (Eichenbaum, 2017), years of reduced sensorimotor exploration may have shaped hippocampal structure through activity-dependent plasticity, potentially limiting later memory capacity. Memory deficits were especially pronounced in children aged 8–11 (Koriakina et al., 2021), a period characterized by hippocampal circuits undergoing major reorganization, synaptic pruning, and connectivity consolidation (Gogtay et al., 2004). Reduced sensorimotor input during this window may have lasting consequences as circuits finalize their formation.

In addition to memory, children with motor disorders demonstrated worse performance in exclusion tasks that measure categorical reasoning to identify a conceptually divergent item within a set. This task directly depends on the integrity of semantic category representations and conceptual relationships. By contrast, performance on Raven’s Progressive Matrices remained intact (Blagovechtchenski et al., 2023). This difference is informative, since it indicates that raw abstract reasoning capacity is preserved, while reasoning processes that draw on the organization of conceptual knowledge are selectively impaired. This pattern is consistent with the EEC prediction that motor restriction disrupts semantically grounded cognition rather than domain-general cognitive capacity.

Deficits also appeared in phonemic fluency tasks (T-word generation), which require strategic search, cognitive flexibility, and cognitive control (Henry, 2015; Marques et al., 2022). The neural basis for these executive impairments may involve the orbitofrontal cortex (OFC). Children with OBPP showed cortical thinning in the right OFC (Kadieva et al., 2024), a region that integrates multimodal sensory and affective information to support reward-based decision-making (Rolls, 2019). The OFC is particularly sensitive to environmental stress and adversity early in life (Hanson et al., 2010; Teicher et al., 2016). In OBPP and AMC patients, reduced environmental exploration and compromised socio-emotional experiences associated with visible disability, peer exclusion, parental overprotection, and limited independent play (Blagovechtchenski et al., 2023) may have contributed to altered OFC development. The resulting structural alterations in OFC may contribute to several of the cognitive deficits we observe. These OFC alterations are consistent with the “embedded” aspect of EEC, with motor disability affecting not only direct body-environment interaction but also fundamentally altering the social and emotional environment, producing wider effects on brain development and cognitive function.

Further evidence consistent with widespread neural differences comes from inter-subject correlation (ISC) analysis during naturalistic video viewing (Ntoumanis et al., 2022). ISC indexes the degree to which the temporal unfolding of whole-brain neural responses is shared across individuals during naturalistic, multimodal stimulation (Hasson et al., 2004). During EEG recording, children passively watched video clips showing real children performing everyday activities and neutral non-human scenes. ISC was computed by measuring the correlation between each child’s ongoing brain activity and the average activity pattern across the group. Patients with motor disorders showed significantly reduced ISC compared with healthy age-matched controls (Cohen’s *d* = 4.36).

Distinctly, the reduced ISC in patients showed no association with the motor-related content of video scenes. When scenes were categorized by movement type (arm, leg, both, or neither), a mixed-effects model revealed that patients’ divergent neural responses were uniform across all categories. Additionally, motor-related content and its interaction with clinical diagnostics did not predict ISC. This uniformity is theoretically important. If motor restriction only affected processing of motor-related content, we would expect selective ISC reduction for hand-action videos with normal responses to leg movements and neutral scenes. The lack of content-specificity challenges straightforward modular theories and suggests a more widespread change in the structure of neural information processing. Hence, we propose that the motor system’s role in cognitive development extends beyond processing movement-related information. Active exploration teaches the brain about the temporal structure of the world, spatial relationships, and object permanence.

This interpretation is supported by control analyses ruling out alternative explanations. One obvious alternative would be problems with attention or engagement, producing noisier neural responses that would mechanically reduce ISC. However, group differences remained significant after controlling for alpha power, a neural marker of attention (Cohen and Parra, 2016), indicating that reduced ISC reflects altered perceptual-cognitive processing rather than attentional differences. No differences in ISC were detected between OBPP and AMC groups, nor did ISC correlate with clinical severity measures, suggesting that the presence of early motor restriction, rather than specific etiology or severity, may drive this widespread neural reorganization.

**Figure 2:**
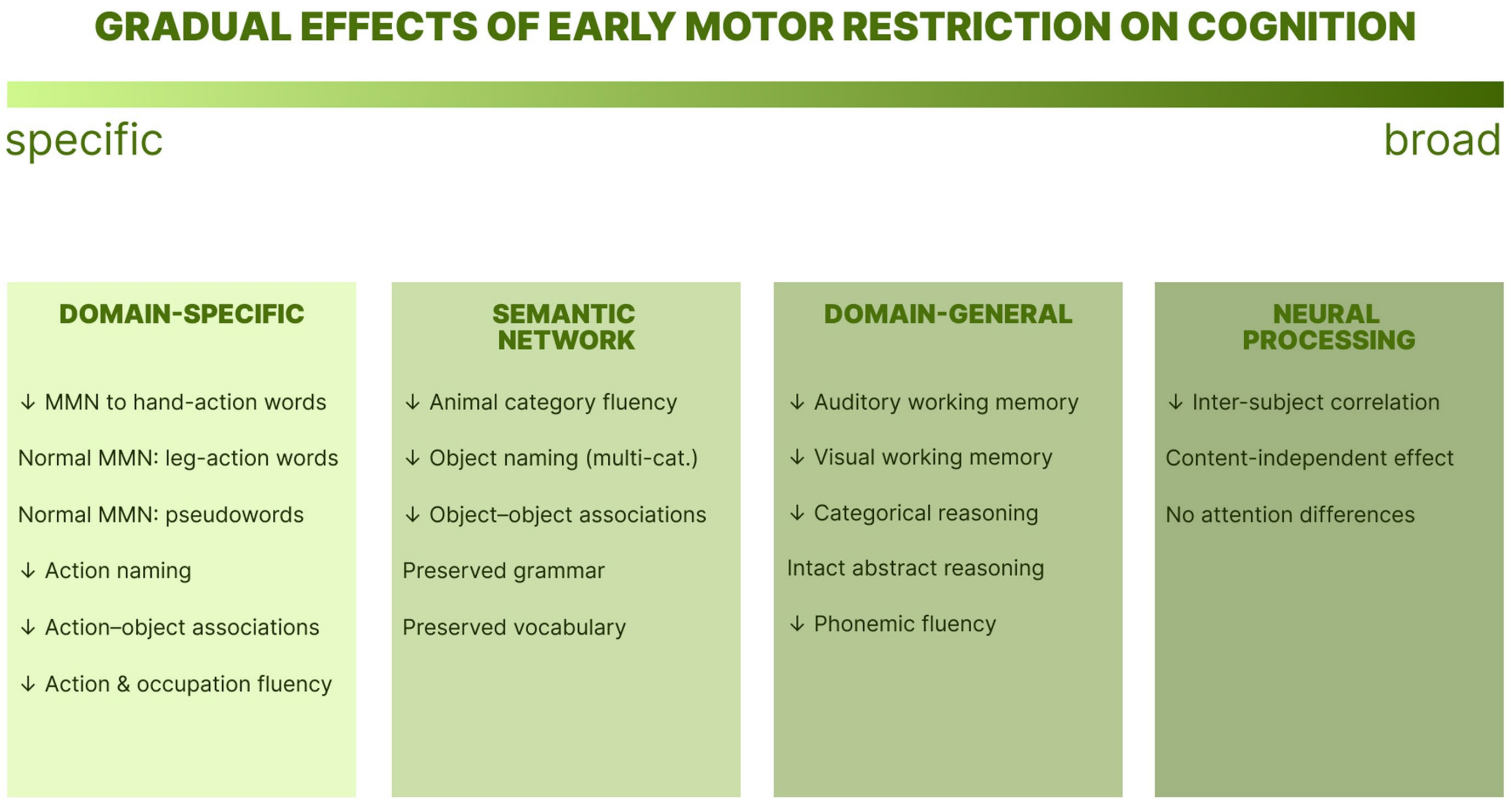
Gradient of cognitive effects associated with early upper limb motor restriction in children with OBPP and AMC. Panels summarize empirical findings at each level of the gradient. Domain-specific deficits in motor-semantic processing (column 1) extend to broader semantic network disruption (column 2), domain-general impairments (column 3), and reduced whole-brain neural response synchrony during naturalistic viewing (column 4).

To sum up, the findings presented across sections reveal a gradient of cognitive alterations spanning from domain-specific deficits in action-semantic processing to widespread changes in neural information processing (Figure 2).

## Discussion

The OBPP and AMC patients offer a rare opportunity to study EEC in a population where bodily interactions with the world are altered prenatally or perinatally. Our findings reveal gradient effects with specific alterations in action-language processing, deficits across broader semantic networks, and wider changes in memory, reasoning, and naturalistic information processing with corresponding structural brain alterations. This pattern suggests that sensorimotor experience does not solely correlate with motor-related cognition—it may also provide the foundation for how distributed cognitive systems evolve during development.

From a theoretical perspective, our findings contribute to ongoing debates about the nature of cognitive development and the role of domain-specific versus domain-general processes. Though the observed simultaneous presence of highly specific deficits (hand-action word MMN) and broad alterations (ISC) may seem contradictory, we would argue that they are compatible within a hierarchical predictive coding framework (Friston, 2005, 2010). Early motor restriction may produce specific representational gaps in lower-level cortical representations, while also altering higher-order predictive models of the environment, consistent with a hierarchical view of embodied development. This view gains additional support from neurophysiological evidence showing that motor cortex involvement in language is both automatic and temporally bidirectional (Shtyrov et al., 2014). This suggests that the motor system is not merely correlated with semantic processing but is an active computational component of it. Early restriction of this system may therefore produce cascading effects, altering not only the representational structure of action concepts, as our MMN data suggest, but also the temporal dynamics of their online activation. However, in our clinical group this interpretation requires direct empirical testing across processing levels within the same individuals.

Additionally, our work underscores the essential interdependence of basic science and clinical application. Children with congenital limb deficiency who receive early prosthetic devices do not show comparable cognitive difficulties (Mano et al., 2023), suggesting that restoring motor-environment interaction during sensitive periods can support typical neurocognitive trajectories. Early surgical or therapeutic intervention to restore motor function, and cognitive-linguistic rehabilitation, may prevent or at least mitigate the debilitating effects we have documented.

Though our work has focused on cognition and brain structure, we have not systematically examined psychosocial and emotional well-being of the patients. These factors are also likely to shape cognitive development. Children with neuromuscular disabilities can face higher rates of anxiety, depression, and peer difficulties. Poor motor skills predict peer problems, which in turn contribute to internalizing symptoms, especially during middle childhood when peer relationships matter most (Mancini et al., 2018). Motor impairment can reduce social participation and quality of life, with cognitive abilities and environmental support playing key mediating roles (Di Lieto et al., 2025). Given the cascading links among motor skills, cognition, and socioemotional competence (Iverson, 2021), longitudinal studies tracking psychosocial stress, peer experiences, and motor-cognitive coupling would provide a richer understanding of these conditions.

Finally, from an EEC perspective (Craighero, 2024), AMC and OBPP present qualitatively distinct developmental challenges. The brain of an AMC patient has never had access to typical prenatal sensorimotor experience and may lack the initial neural architecture upon which action-semantic representations are built. The fact that our studies found no significant cognitive differences between these groups is therefore ambiguous. It could reflect an insensitivity to timing of onset, or insufficient statistical power to detect real differences. Similarly, OBPP is strictly unilateral, potentially allowing compensatory sensorimotor grounding via the intact arm (Hahamy et al., 2015). AMC on the other hand produces bilateral restriction, so one would expect AMC to be associated with more pervasive cognitive deficits. Yet no significant differences between the groups were observed. Future studies designed specifically to contrast AMC and OBPP would be needed to address these issues directly.
